# Proceedings of the American Institute of Homœopathy

**Published:** 1894-09

**Authors:** 


### DEFORMITIES AND DISEASES OF CHILDREN.

In the Section in Pædology of the American Institute of Homœopathy, Dr. Millie J. Chapman's paper on "Prevention of Deformities" was especially valuable. It treated of not only malformations and monstrosities as found, but their cause and prevention. Dr. Chapman's old study of the adaptability of persons entering matrimony were touched but lightly in the paper, but were afterward brought out in the discussion. The statement was made that it was the law of heredity that deformities commonly reappear for four or five generations and seldom disappear altogether in ten or twelve. Parents produce children having similar natures and tendencies as their own. Then by restoring the health of the parents it generally remedied the deformities of the children. Many deformities were attributable to the emotions of the mother. A number of cases were given which showed that where the tendency was proven to be in the direction of deformed offspring it was corrected by proper methods, not always medical and seldom surgical.

Dr. Charles A. Gale's paper on "Tubercular Meningitis" showed that this disease must be recognized early for the physician to do anything to stay its progress. He claimed that under homœopathic treatment the disease could be cured and many children who would otherwise die early with tubercular meningitis were cured and grow to powerful health. This view was taken by several others who cited cases and results in their own practice.

Dr. William W. Van Baun, of Philadelphia, editor of the *Hahnemann Monthly*, read a paper on "Scurvy in Infancy and Childhood." He called attention to the general increase of the disease in America, among the children of the wealthy as well as those of the poor. The principal cause, he claimed, was the present free use of artificial or proprietary food, although anything given in the line of nourishment overtaxing the infantile digestive system, coupled with a deficient supply of fresh animal or vegetable product, would tend to develop scurvy. The treatment of the condition depended altogether upon the application of proper dietary means and a plentiful supply of fruit acids.

"Cholera Infantum," by Dr. C. H. Thomas, of Cambridge, Mass., was the title of a paper of particular interest to the general public as well as the physicians. The speaker said:

"This is a disease which goes hand in hand with warm, sultry weather, especially prevalent during dog days, when the atmosphere day and night is surcharged with humidity; and as that period is rapidly approaching, a discussion of symptomatology, dietary and several well-tried drugs applicable to this affection is certainly a timely one. Owing to the sudden manifestations and rapid tendency deathward, no opportunity should be lost in bringing all our armament to bear directly upon the morbid process and its causes, exciting or otherwise. Germicides, when used as such, better be put into a separate class, and labeled homicides or infanticides; but when well potentized and clearly indicated, are the remedies most frequently used and the most reliable.

"In seeking for the many causes it is well to keep in mind the source of the milk supply, the character of the food furnished the animals and their surroundings, its age and absence of so-called milk preservatives which are in general use among dealers during the summer months; condition of nursing bottles, utensils, and water used in the preparation of food and the manner in which it is fed to the infant. Many cases can be traced to the fact that the babe is early put to bed with a full bottle and allowed to draw upon the lacteal fount until morning, necessarily feeding on sour milk and the products of fermentation. Near large cities many of the dairy cows are fed on swill or brewery grains and refuse from produce markets; kept in close quarters, poorly lighted and ventilated, with large heaps of excrement piled up, immediately under the only inlets for pure and fresh air. It will certainly repay any and all physicians having nursing bottle fed infants under their care to devote some of their energies to the location and character of their food supply and not wait until their charges are suddenly taken sick, prevention being vastly superior to cure. I have been obliged by clinical experience to place the most dependence upon Arsenite of Copper, Helleborus-nig., Colocynthis, Merc-corr., and Calcarea-phos., and believe a majority of cases will respond to these remedies.

"The following case will possibly serve to illustrate what the two drugs first named can and did accomplish. Case—Baby, age, four months, light complexion, scrawny, poorly nourished, was taken with cholera infantum July, 1893. A consultation of old-school physicians, after treatment by their method, resulted in prognosis—'Fatal within three hours; everything possible has been done.' In this condition the patient was turned over to me for what was most probable, the 'certificate of death.'

"The first thing done was to remove all clothing, thoroughly sponge the little one's body in tepid water, remove all local spicy applications, open all the windows on that floor for fresh air, remove the patient from before a hot fire in the kitchen stove, clearing the room of all but one person and administering Cuprum-ars., 6x, every half hour. After the third exhibition of the remedy, the convulsions ceased, with the other symptoms substantially improved. Another visit was made at midnight; child asleep; no discharges since 7 P. M.; was fed at 8 P. M. on malted milk, and this was the only food used during and after convalescence, and filling all requirements. The next day, Helleborus-nig., 6x, was exhibited hourly for three hours, then discontinued, as all demands for it had ceased. The next day (third) the child was taken to the seashore in an open carriage, and remained there all day, sleeping quietly most of the time, returning at sunset with the elixir of life manifested in all its glory. The boy still lives, healthy and robust, with every prospect of a bright future, and the family and neighbors firm converts to Homœopathy and its possibilities."

Other interesting papers read during the session of the bureau were: "Hahnemann's Doctrine of Psora in the Treatment of Disease in Children," by Dr. W. Bœricke; "Barlow's Disease," by Dr. Martin Deschere; "The Paralyses of Diphtheria," by Dr. O. E. Janney, and "The Sexual System and Procreation," by Dr. J. C. Nottingham. The next meeting of the bureau will be held Wednesday morning.

---

### HYPNOTISM IN SURGERY.

A very interesting paper on "Hypnotism in Surgery" was read by Dr. Henry W. Roby, of Topeka, Kan., before the Surgical Section of the American Institute of Homœopathy. He began with a reference to the occult forces and their phenomena and the uses, mostly grotesque or evil, to which they had been put, which resulted in their discountenance by Church and State everywhere. Jugglery and jingoism had to be shaken off and the field cleared for the real work. He spoke of chemical anæsthesis as a great discovery, but said that here was a force that could lull pain without putting the patient in peril, as does the use of Chloroform or Ether. He spoke of the magnetizing of horses in Austria before shoeing them, and the wonderful power of horse trainers who used this odylic force. He reviewed the early use of hypnotism by surgeons and classified its phenomena as having three states—the cataleptic, lethargic, and somnambulistic.

He related the mysteries of the occult forces and the wonderful things done by Oriental sorcerers.

### CHARCOT AND LOMBROSO.

Speaking of the modern use of this power, he said :

"In France great hospitals for nervous diseases have been established, in which hypnotism plays a large *rôle* in the curative proceeding. In the great Salpetriere in Paris Charcot has achieved a world-wide reputation as a medical hypnotist, and has achieved some of the most wonderful cures, while in the great hospital at Nancy Liebault has made many astonishing cures of nervous affections.

"Lombroso, of the University of Turin, has also achieved great reputation, not only in the hypnotic cure of nervous diseases but of drunkenness and crime.

"In an excellent work on magnetism written forty years ago James Victor Wilson says :

"'When Sir William Bell wrote his treatise on *The Human Hand*, and exhibited its admirable and ingenious mechanism, he left altogether unnoticed by far the most wonderful and adorable feature of its structure, its power of transmitting at the fingers' ends the life forces of the system to the alleviation of pain, and even the eradication of disease in others, its power of throwing strong men into a torpor in which the most frightful surgical operations can be performed without pain.'

"Long before the discovery of Sulphuric Ether French and other surgeons were using hypnotism as an anæsthetic agent. Cloquet, Broca, and Velpeau (whom the French call the king of surgeons) amputated arms, breasts, and legs under its benign sleep, and Esdaile has performed over a thousand surgical operations on hypnotized Hindoos in the great hospital at Calcutta. In all our great cities there are men who do a large amount of medical and surgical work by means of this benign agency. To most of us this great resource of medicine and surgery is practically unknown. One value in the study of anything new is to wake up the mind and send it off in the chase after knowledge. That constitutes a large amount of the good in all conventions and all assemblies, in all sermons and lectures, in all dramas and concerts and oratorios, and I present this paper in the hope that it may send your minds out in search of that which does not yet lie clearly revealed to the minds of men.

"As many great powers for good are also great powers for evil, so it is with this marvelous agency. Its maladroit use has done much harm in the world. So has the maladroit use of Quinine, electricity, and the surgeon's knife. But science is now taking it up and turning on the bright light of critical investigation, and its beneficent mission on earth is being more clearly demonstrated, and its dangerous operations are being repressed by the iron hand of the law.

"Psychological societies are now organized all over the world, and thousands of scientists are making critical study of the psychic forces, and already a voluminous literature is extant on the subject. Somewhat recently there has been issued from the press of McClurg & Co., of Chicago, a very excellent work on *The Law of Psychic Phenomena*, by Thompson J. Hudson, in which the author claims to give the scientific basis and the working formula of all psychic phenomena, and he supports that claim with a great array of facts and cogent reasoning. That work should be in the hands of every scientist who desires to know what may be learned concerning odylic force.

"I bring the subject to your attention at this time in the hope that you who practice surgery may use and study hypnotism more extensively in practice than is now done. In the last two years I have used it to a considerable extent in surgical work; and in the cases where I can make it available, I find it much more satisfactory than chemical anæsthesia. It puts no patient in peril of his life, as do Chloroform and Ether. It takes little or no longer to obtain the anæsthetic state and the patient can be awakened from it instantly, or he can be left hypnotized against the sense of pain for an indefinite period, while all his other faculties and sensibilities are left in a normal state. He can thus be tided over that otherwise always much dreaded period of post-operative pain, which all sensitive surgeons feel keenly in sympathy with their patients."

The paper was received with attention and aroused much discussion.

---

### PATENT FOODS.

In the Section in Pædology of the American Institute, there was a discussion upon patent foods. W. W. Van Baun was Chairman of the Section, and Charles A. Gale, Secretary. A paper upon "Barlow's Disease" was read by Secretary Gale. The paper was written by Martin Deschere, M. D., of New York. He describes the disease as a peculiar condition in children which was difficult to classify under the head of any positive pathological state. Some describe it as an acute rachitis, others as scurvy, and again it has been thought to be a combination of purpura with rachitis. Dr. Thomas Barlow, of London, gave the first positive anatomical description of this affection. During the first two years of life the affection was characterized by exceedingly painful swelling in various bony regions, with symptoms of rachitis. Anatomically the swellings were caused by hemorrhages. The disease was most frequent in children ranging in age from six to eighteen months. The bones became tender and great pain was inflicted when any attempt was made to move the child. The writer then prescribed cures for the disease. Concluding he said: "There seems no greater surviving fallacy current in medical practice than the feeding of tender infants upon the patent productions of medical firms."

Chairman Van Baun then read a highly interesting paper upon "Scorbutus in Infancy and Childhood." The paper was discussed at length. Dr. Gale said that in his opinion the cause of scurvy in children was improper nourishment, causing indigestion. The giving of food to children at all hours was the cause of a great deal of trouble. If a child were brought up systematically and taught to eat food at proper hours better men and women would be reared.

Dr. McClelland told of a case treated by him recently where it was necessary to amputate a child's foot in an extreme case of scorbutus.

The Section in Pædology then adjourned *sine die.*

---

### RELATION OF CLIMATE AND ALTITUDE TO INSANITY.

[Proceedings of the American Institute.]

Dr. H. R. Arndt, a specialist of San Diego, Cal., read an able paper upon the relation of climate and other conditions to insanity. The question was one of great difficulty, he said, and required years of study by experts to obtain correct results. A reason for the large proportion of insane persons in the West could be said to be the excessive brain work engaged in by many. He described the great gold excitement in California, and the real estate booms of a later day with relation to their effects upon the minds of the children of those who struggle with fortune, many of whom had gained riches in a day. He felt satisfied from personal observation that a large proportion of the inmates of the insane asylums of California came from the middle valleys of the State, many of whom lived in isolated spots and had no intercourse with the outside world. Many of these people fell into a state of apathy, afterward resulting in delusion and insanity.

Dr. Campbell, President of the San Bernardino Insane Asylum, spoke to the question of the causes of insanity. He said that although it was the prevailing opinion that insanity was more common in California than in other parts of the country, it would be soon demonstrated statistically that the proportion of insane was not greater than in other States. He knew from personal experience that a part of the inmates of the asylums had lived in the State only a few months in the hope of regaining lost health. In the San Bernardino Asylum there were many natives of the State, men and women of Spanish and Mexican blood. The proportion as to sex was two to one, the larger number being men.

Dr. H. P. Skiles, of Chicago, said that in his opinion insanity was not caused so much by brain troubles as by bodily ills. Under the homœopathic treatment, as practiced in several hospitals for the insane, those affected were put to bed and treated as sick persons. He believes that melancholia could be cured by rest and local treatment.

### THE USE OF MERCURY.

After the American Institute adjourned, the Sections in Materia Medica and Sanitary Science met. Professor Allen spoke on "Mercury" before the section. He told how the drug had been extensively used for a hundred years, and explained the poisonous influence it exerts. Its action on the liver and the kidneys caused these organs to become inflamed. It arrested the secretion of bile in the liver and led to the destruction of the organ. It had an almost similar action on the kidneys. Mercury caused ulcerations all through the body. It also destroyed the blood. Wounds and fractures would not heal under the influence of Mercury.

Mercury increased the perspiration and in all the cases of poisoning by its use the patients were worse at night. Mercury attacked the long bones, but not the flat bones, and destroyed them.

Professor Allen went on and described the uses of the drug at length, and at the close of his address he was heartily applauded.

